# FourFlow - open source code software for quantification and visualization of time-resolved three-directional phase contrast magnetic resonance velocity mapping

**DOI:** 10.1186/1532-429X-14-S1-W14

**Published:** 2012-02-01

**Authors:** Einar Heiberg, Christopher Green, Johannes Toger, Andreas M Andersson, Marcus Carlsson, Hakan Arheden

**Affiliations:** 1 Department of Clinical Physiology, Lund University, Lund, Sweden; 2 Department of Numerical Analysis, Centre for Mathematical Sciences, Lund University, Lund, Sweden

## Summary

We have developed an open source software for quantification and visualization of 4D PC-MR data that enables development of new quantitative analysis tools.

## Background

Time-resolved three-directional phase contrast magnetic resonance velocity mapping (4D PC-MR) has the potential to be a powerful modality for studying normal physiology and pathophysiology. However, a wider application of 4D PC-MR is hampered by a lack of methods for quantitative analysis. Currently available software packages for visualization of 4D PC-MR do not allow for modifications or additions by users. This limits innovation of new quantification tools, and limits the possibilities to answer specific clinical and physiological questions. Therefore, the aim of this study was to develop a comprehensive open source software for quantification and analysis of 4D PC-MR data that includes the possibility to develop and add new quantitative analysis methods.

## Methods

As a first step we identified common visualization tasks, and listed required features for quantification and visualization of 4D PC-MR. We reviewed existing open source projects that could be used as a starting platform. ParaView (www.paraview.org) was found to be the best candidate. A new user interface was implemented on top of ParaView. The Segment (segment.heiberg.se) was used to read and process DICOM images.

## Results

The software project was named FourFlow. The figure shows the user interface. Quantification tools currently implemented in the software are flow and velocity quantification and quantification of kinetic energy. The software has an interface that allows researchers to write extensions and develop new quantitative tools. Fourflow includes standard visualization techniques such as particle trace (forward and backward), streamline analysis, clip planes, isosurfaces, volume rendering, and display of anatomical Cine images. As an example of possible software extensions, we implemented the new analysis method Volume Tracking that allows tracking of selected volumes and study how they move and deform (Toger et al, BMC Medical Imaging 11:10, 2011). As another example of software extension we implemented vortex core detection. FourFlow will be made freely available to researchers at the completion of the project.

**Figure 1:**
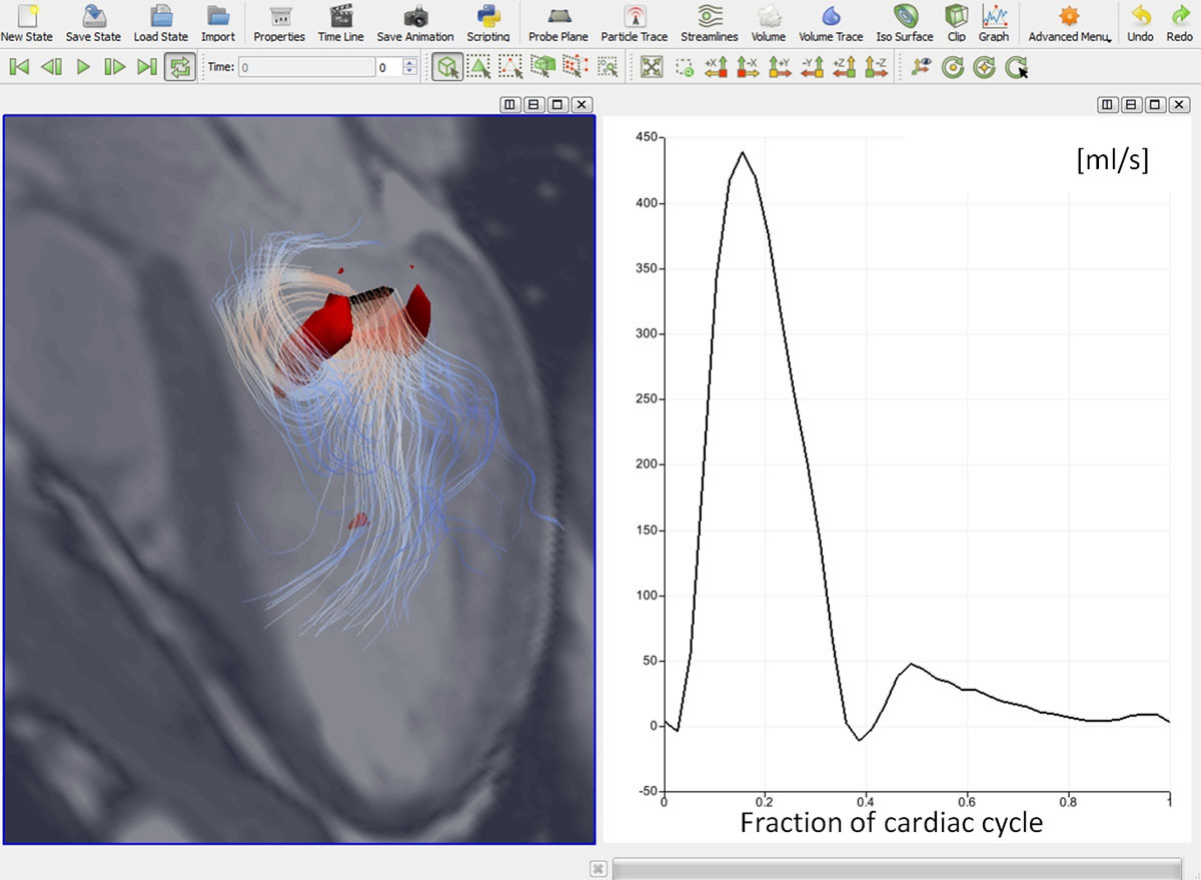
FourFlow user interface. Left panel shows a three-chamber view for orientation, and automatically detected vortex cores (red) in early diastole. Streamlines are released downstream from the mitral valve. The right panel shows flow quantification in the proximal ascending aorta from 4D PC-MRI.

## Conclusions

This project is the first open source software specifically developed for quantitative analysis of 4D PC-MR. By allowing user extensions and modifications the software will facilitate research and development of new quantitative analysis tools.

## Funding

Swedish research council 2008:2949, 2008:2461. Swedish Knowledge Foundation, Region of Scania.

